# Psychotherapeutic support peculiarities’ in palliative care structure for cancer patients

**DOI:** 10.1192/j.eurpsy.2021.1330

**Published:** 2021-08-13

**Authors:** B. Mykhaylov, V. Postrelko, O. Galachenko, A. Vasilyieva

**Affiliations:** 1 Psychiatry, National Academy of Postgraduate Education, Kiev, Ukraine; 2 Department Of Medical Rehabilitation And Medical Social Expertise, Vinnytsia National Medical University M. Pirogov, Vinnytsia, Ukraine; 3 Psychiatry, Donetsk State Medical University. M. Gorky, Liman, Ukraine

**Keywords:** palliative care, hospice, the cancer patients, psychotherapy.

## Abstract

**Introduction:**

PPalliative medicine is aimed at achieving the best possible in a particular situation the level of quality of life of the patient.

**Objectives:**

The reaction of grief is one of the strongest and most painful human experiences.

**Methods:**

There were 120 cancer patients observed. The reaction of grief consists of 4 stages: shock and protest - numbness, disbelief and acute dysphoria; absorption - acute longing, search and anger; disorganization - a sense of despair and acceptance of loss and decision.

**Results:**

The initial reaction of grief - shock, emotional numbness and disbelief. The excitement is most pronounced within about two weeks, followed by symptoms of depression, which reach its peak 4-6 weeks. Eventually, the former intense pain of severe loss begins to subside. In addition to the reaction of grief, there is a pathological, which is divided into suppressed (inhibited), delayed (delayed) and chronic. The role of the psychotherapist at this stage is to provide psychological support and assistance to both the patient and his environment to cope with this situation.

**Conclusions:**

The principle of the concept of palliative care is the need to ensure the psychological comfort of the patient.

